# Factors influencing age of common allergen introduction in early childhood

**DOI:** 10.3389/fped.2023.1207680

**Published:** 2023-07-11

**Authors:** Michael Marget, Yamini V. Virkud, Wayne G. Shreffler, Victoria M. Martin, Qian Yuan

**Affiliations:** ^1^ Food Allergy Center, Massachusetts General Hospital, Boston, MA, United States; ^2^ Division of Pediatric Allergy and Immunology, Massachusetts General Hospital, Boston, MA, United States; ^3^ Division of Pediatric Allergy & Immunology, University of North Carolina, Chapel Hill, NC, United States; ^4^ Department of Pediatrics, Harvard Medical School, Boston, MA, United States; ^5^ Division of Pediatric Gastroenterology, Hepatology and Nutrition, Massachusetts General Hospital, Boston, MA, United States

**Keywords:** solid food in infancy, allergen introduction, peanut, peanut introduction, egg

## Abstract

**Objectives:**

We evaluated factors influencing the timing of allergen introduction in the U.S., including updated peanut introduction guidelines.

**Study design:**

The Gastrointestinal Microbiome and Allergic Proctocolitis (GMAP) study is a prospective observational cohort in suburban Massachusetts. Infants' caregivers enrolled between 2014 and 2017, and they reported when they introduced common allergens to their child. Multivariable linear and survival regression analyses were used to examine factors influencing time of introduction of allergens.

**Results:**

By 9 months, children old enough to be potentially affected by NIAID's 2017 peanut introduction guidelines were more often introduced to peanut than children enrolled well before guidelines publication [54% vs. 42%, OR: 1.63, CI: (1.03, 2.57), *P *= 0.03]. At any given time, Black children were 73% [HR: 0.27, CI: (0.11, 0.69), *P *= 0.006] less likely to be introduced to peanut as early as White children. Asian children were, respectively, 36% [HR: 0.64, CI: (0.47, 0.86), *P *= 0.003] and 26% [HR: 0.74, CI: (0.55, 0.97), *P *= 0.03] less likely to be introduced to peanut and egg as early as White children. A first child was 27% [HR: 1.27, CI: (1.04, 1.56), *P *= 0.02] more likely to have been introduced to peanut earlier than a non-first child. There was no association between age of introduction and sex, gestational age, family history of food allergy, or other allergic comorbidities.

**Conclusion:**

Updated introduction guidelines, race, and birth order all influenced earlier introduction of peanut. Further studies to evaluate current practices for allergen introduction with a focus on potential disparities are needed.

## Introduction

IgE-mediated food allergy affects approximately 8% of children in the United States (1). While treatment options beyond avoidance are increasing, food allergy continues to present a significant burden for those affected (2). Recent studies have provided evidence that the early introduction of allergens during infancy can help prevent the development of IgE-mediated food allergy (3–6). Originally published in 2015, the Learning Early About Peanut Allergy (LEAP) study found that early peanut consumption beginning before 11 months of age led to a reduction in the development of peanut allergy at 5 years compared to infants who avoided peanut before that time (7). A meta-analysis of 5 randomized-controlled trials evaluating the effect of early egg introduction during infancy on later egg allergy development found that egg introduction from 4 to 6 months of age was associated with a reduced risk of egg allergy compared to later introduction (8). These led to the new Addendum Guidelines for the Prevention of Peanut Allergy in 2017 published by the National Institute of Allergy and Infectious Diseases (NIAID) recommending peanut introduction at or before 6 months for infants at higher risk of peanut allergy development (9). The American Academy of Pediatrics in 2019 recommended early introduction of peanut for children at a high or moderate risk for developing peanut allergy (3). In 2021, some leading international allergy organizations recommended introducing egg and peanut around 6 months of age for all infants (10). The creation of new guidelines is a key step towards improving the early introduction of common allergens in infancy, however the adoption of clinical guidelines is based on a variety of complex factors and often does not occur rapidly (11).

While there has been extensive research on the potential benefits of early introduction of common allergens, there has been less focus on characterizing their present age of introduction in the U.S. A Canadian cohort study enrolling from 2008 to 2012 found that 45.1% of their cohort was introduced to peanut by 12 months (12). An Australian cohort study found the median age of peanut and egg introduction in their cohort was 6 months of age for both foods (13). They also analyzed peanut introduction before and after Australia updated its peanut introduction guidelines in 2016 and found that after guidelines publication 88.6% of infants were introduced to peanut by 12 months of age compared to 28.4% from an earlier Australian cohort (13). We sought to evaluate common allergen introduction practices of children in our healthy infant cohort, the Gastrointestinal Microbiome and Allergic Proctocolitis study (GMAP) (14). GMAP prospectively surveyed families with respect to their feeding practices across the first several years of age, and we examined whether the new guidelines had an impact on those practices. We also set out to evaluate what factors may be associated with earlier allergen introduction.

## Materials and methods

The GMAP Study is a prospective observational cohort study of healthy children followed in a Massachusetts suburban pediatric practice starting with their first well-child visit (14). Infants were enrolled between March 2014 and February 2017, and the current median age of the cohort is 6 years. Enrollment was consistent throughout the study. The Massachusetts General Brigham Institutional Review Board (IRB) approved the GMAP study, and all parents of enrolled children gave written informed consent.

At every well-child visit (1 week, 2 week, 1 month, 2 month, 4 month, 6 month, 9 month, 12 month, 18 month, 24 month, 36 month), parents were administered a questionnaire assessing the infants' feeding practices including whether they were introduced to any solid foods, and which foods (including common allergens) had been introduced. These data were acquired prospectively through automated survey invitations using an electronic data capture system (Research Electronic Data Capture, REDCap, hosted by Mass General Brigham) approved for use by the IRB. To capture missing data, and to account for the lag time between introduction and the next well-child visit survey administration, parents were also asked once after their child turned three to retrospectively report the age of introduction of common allergens, and the earliest reported age of introduction was used. We chose to focus on introduction of peanut and egg given the change in introduction recommendations for these foods over the last decade. For comparison, we also analyzed wheat introduction given its relatively common early introduction and unchanged introduction guidelines. To assess food introduction practices before and after NIAID's 2017 peanut introduction guidelines, the cohort was split into a pre-guidelines group who were 6 months or older at the time of publication (“pre-2017 guidelines”) and a post-guidelines group (“post-2017 guidelines”) who were the remainder of the cohort.

All analyses and figures were produced using the programing language R (4.0.2) (15). We used *χ*^2^ and Fisher's exact tests to explore relevant associations between age of introduction and variables of interest. Of note, we selected 9 months of age as the time point to characterize if post-2017 guidelines children were introduced to allergens earlier than pre-2017 guidelines children. We chose 9 months of age because the guidelines target 6 months of age, and a change made at 6 months would be reflected in the next survey completed at 9 months of age. We also used a Cox proportional hazards model in exploratory survival analysis to examine the association between variables of interest and the age of introduction of allergens. Multivariable analysis was adjusted for sex, race, ethnicity, birth order, gestational age, eczema, FPIAP, IgE-mediated food allergy, and family history of food allergy.

## Results

**Figure 1:**
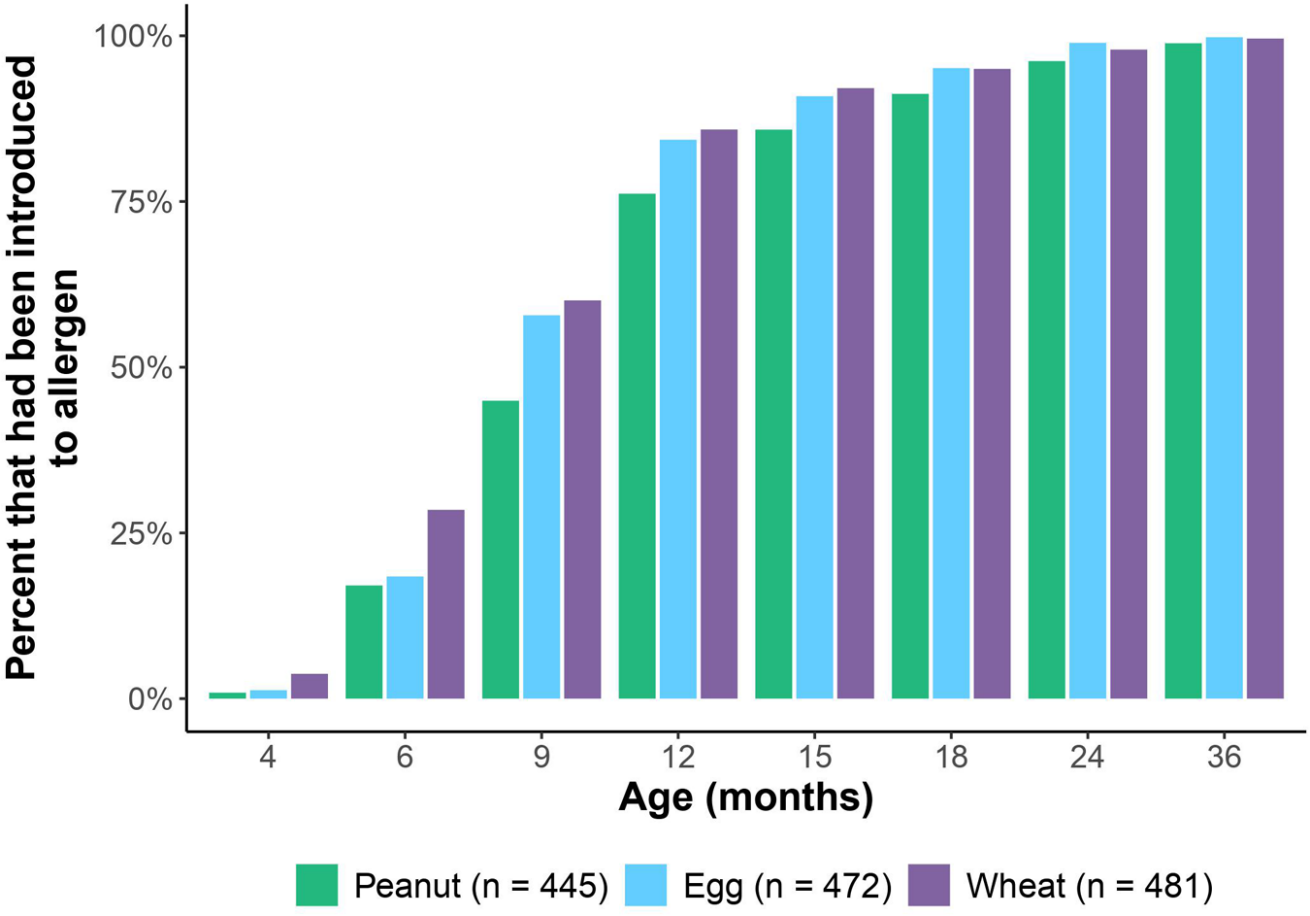
Percent of cohort at each well-visit timepoint who had been introduced to peanut, egg and wheat.

Of the 903 subjects used in the full GMAP cohort (14), 494 had adequate allergen introduction data for analysis. This sub-cohort did not significantly differ from the larger cohort with respect to sex, race, ethnicity, birth order, gestational age, FPIAP, IgE-mediated food allergy, or family history of food allergy. The sub-cohort did differ by having a higher proportion of children with eczema compared to the larger GMAP cohort (50.6% vs. 43.2% respectively). The median age of solid food introduction was 6 months [range: 4–13]. Regarding the allergens of interest, the median age of introduction was 12 months for peanut [range: 3–67], 9 months for egg [range: 4–37], and 9 months for wheat [range: 3–37]. Of note, 45% of the cohort were introduced to peanut by 9 months and 76% were introduced by 12 months. By 18 months of age, all three allergens had been introduced in at least 90% of the cohort (Figure 1).

**Figure 2:**
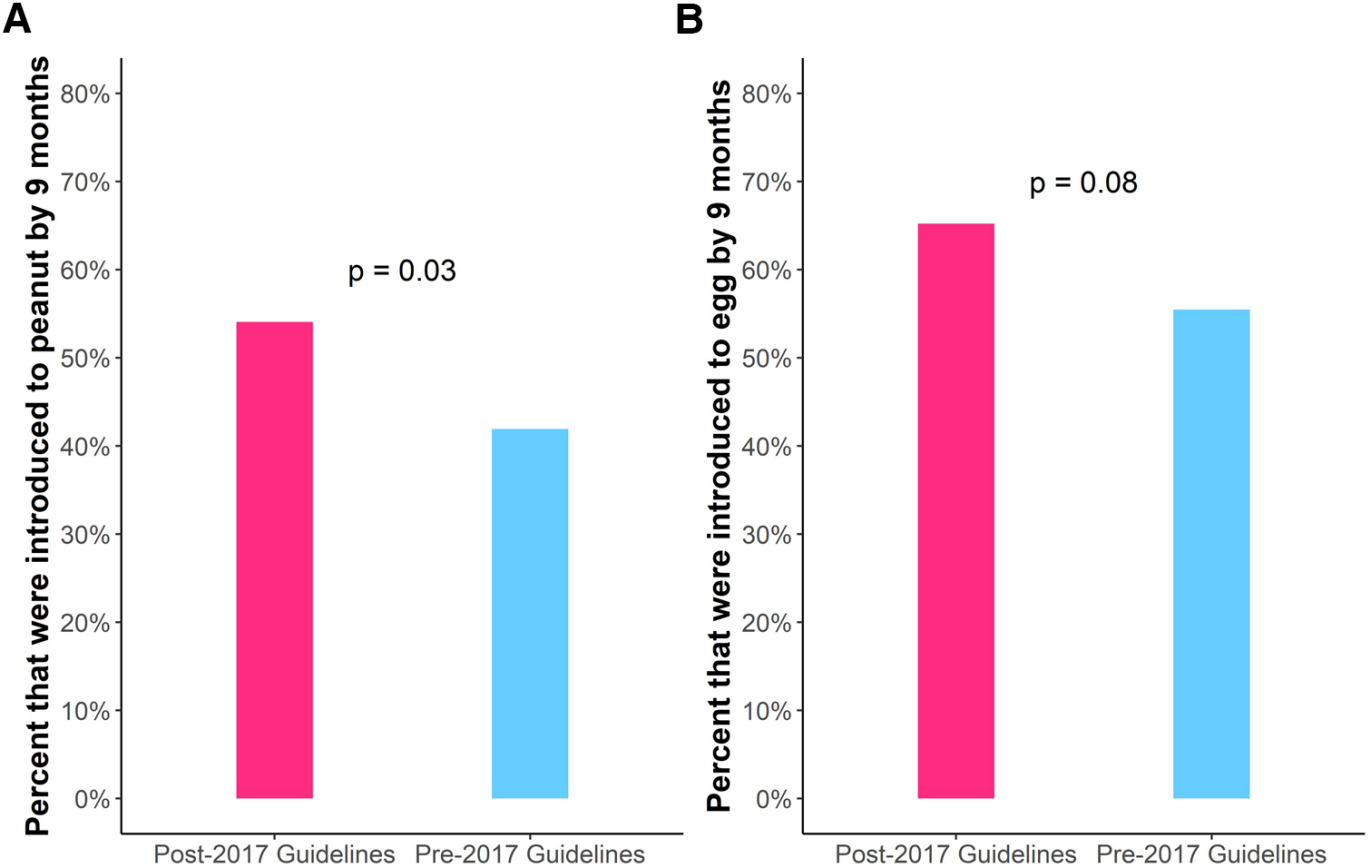
Percent introducing peanut (**A**) and egg (**B**) by 9 months of age separated by age in relation to publication of NIAID's 2017 peanut introduction guidelines. Post-2017 guidelines infants were more likely to have been introduced to peanut (*P* = 0.03) than pre-2017 guidelines infants.

We then examined the age of introduction in children who were in the pre-2017 guidelines (*n* = 377) and post-2017 guidelines groups (*n* = 117) to examine introduction practices before and after publication of NIAID's 2017 peanut introduction guidelines. The pre-2017 guidelines and post-2017 guidelines groups did not differ significantly with respect to the above-mentioned demographic factors (Table 1). Post-2017 guidelines infants were statistically significantly more likely to have been introduced to peanut by 9 months of age compared to pre-2017 guidelines infants [54% vs. 42%, OR: 1.63, CI: (1.03, 2.57), *P* = 0.03] (Figure 2). More post-2017 guidelines infants were introduced to egg by 9 months than pre-2017 guidelines infants, but this was not statistically significant [65% vs. 55%, OR: 1.50, CI: (0.95, 2.40), *P* = 0.08] (Figure 2). There was no difference in age of introduction of wheat by 9 months. To address the continuous effects of time outside the nine-month period we conducted survival analysis and found there was no statistically significant difference in the time to introduction of peanut, egg, or wheat (Supplementary Table S1). For peanut, we noted a decrease in delayed introduction (introduced to peanut later than 24 months of age) in post-2017 guidelines infants (0.9%) vs. pre-2017 guidelines infants (4.8%) using survival analysis though this was not statistically significant [OR: 5.52, CI: (0.84, 233.96), *P* = 0.08].

**Table 1:** Comparison between pre-2017 guidelines and post-2017 guidelines groups.

	Pre-2017 guidelines	Post-2017 guidelines	*P*
Overall *n*	377	117	
Female (%)	170 (45.1%)	62 (53.0%)	0.2
Race	–	–	0.1
White (%)	273 (73.2%)	75 (65.2%)	
Asian (%)	56 (15.0%)	28 (24.3%)	
Black (%)	6 (1.6%)	2 (1.7%)	
Other Race (%)	38 (10.2%)	10 (8.7%)	
Hispanic or Latino (%)	17 (5.0%)	6 (5.5%)	1.0
Gestational Age	–	–	0.5
>37 Weeks (%)	343 (91.0%)	105 (89.7%)	
33–37 Weeks (%)	31 (8.2%)	12 (10.3%)	
25–32 Weeks (%)	3 (0.8%)	0 (0.0%)	
Eczema (%)	190 (50.4%)	60 (51.3%)	1.0
First Child (%)	172 (45.7%)	65 (55.6%)	0.08
IgE-FA (%)	28 (7.4%)	8 (6.8%)	1.0
Family History of IgE-FA (%)	68 (18.0%)	13 (11.1%)	0.1
FPIAP (%)	66 (17.5%)	22 (18.8%)	0.9
Introduced peanut by 9 months (%)	140 (41.9%)	60 (54.1%)	0.03
Introduced egg by 9 months (%)	198 (55.5%)	75 (65.2%)	0.08
Introduced wheat by 9 months (%)	217 (59.6%)	72 (61.5%)	0.8

**Figure 3:**
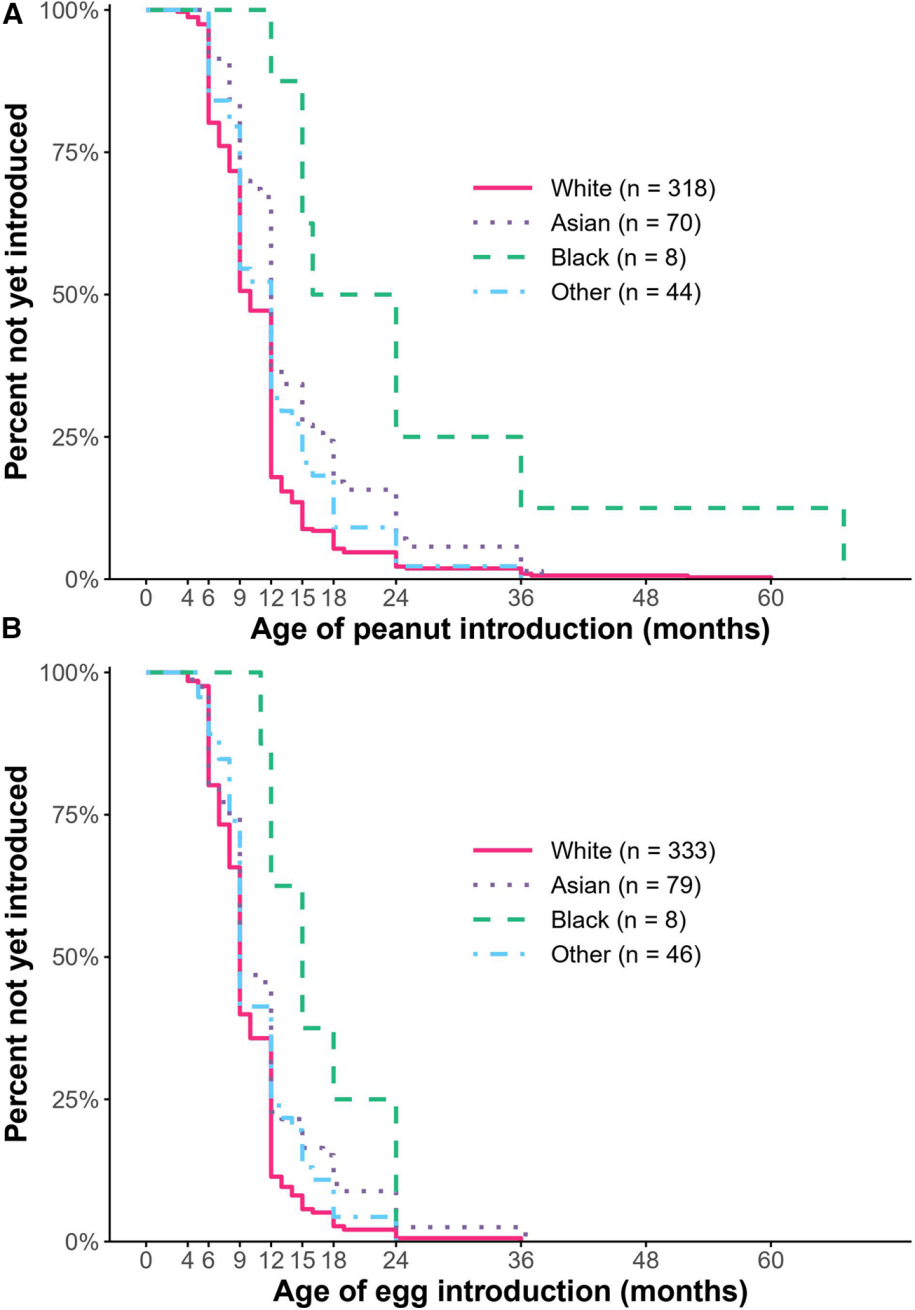
Kaplan–meier curve depicting the percent who had not yet introduced peanut (**A**) or egg (**B**). Compared to White children, Black children were less likely to have been introduced to peanut and egg, and Asian children were less likely to have been introduced to peanut as early (*P* < 0.05).

Using multivariable survival analysis to assess the association of demographic and allergic covariates with time to introduction of allergens (Supplementary Table S1), we found that race and birth order were significantly associated with the age of introduction of some allergens, even after adjusting for all the other factors including 2017 guidelines status. At any particular time over the first six years of life, Black children were 73% less likely [HR: 0.27, CI: (0.11, 0.69), *P* = 0.006] and Asian children were 36% less likely [HR: 0.64, CI: (0.47, 0.86), *P *= 0.003] to have been introduced to peanut as early as White children (Figure 3). A first child was 27% more likely to have been introduced to peanut earlier than a non-first child [HR: 1.27, CI: (1.04, 1.56), *P* = 0.02].

Regarding egg, Black children were 52% less likely [HR: 0.48, CI: (0.21, 1.09), *P *= 0.08]; and Asian children were 26% less likely [HR: 0.74, CI: (0.55, 0.97), *P* = 0.03] to have been introduced to egg as early as White children (Figure 3). Of note, the association between delayed introduction of egg among Black children was significant using univariable survival analysis, but not multivariable analysis. There was no significant difference in egg introduction based on birth order. Regarding wheat introduction, Black children were 68% less likely [HR: 0.32, CI: (0.13, 0.79), *P* = 0.01] and Asian children were 28% [HR: 0.72, CI: (0.54, 0.95), *P* = 0.02] less likely to have been introduced to wheat as early as White children. There was no association observed between age of introduction of peanut or egg and sex, gestational age, family history of food allergy, or other allergic comorbidities.

## Discussion

We found that the median age of peanut, egg, and wheat introduction in our healthy infant cohort between 2014 and 2017 was 12, 9, and 9 months respectively, and that after publication of NIAID's 2017 peanut introduction guidelines, children were more likely to have been introduced to peanut by 9 months of age. Still, 46% of the post-2017 guidelines cohort had not been introduced to peanut by 9 months of age and almost a quarter of the whole cohort had not been introduced by 12 months of age. We did not find a statistically significant difference in egg introduction by 9 months between groups, but this is unsurprising given that there were no U.S. guidelines to suggest earlier egg introduction during the study period. We found that first-born children were more likely to have been introduced to peanut early compared to children with older siblings. We hypothesize this may be due to parents with several children following outdated guidance from visits with their older children.

We also found that Black and Asian children in our cohort were introduced to peanut significantly later than White children. Similarly, another study of children in the United States enrolling 0–12 year-old children found that White children were more likely to have been introduced to peanut before 6 months and less likely to have been introduced after 11 months compared to Black children (16). With respect to egg introduction, Black and Asian children in our cohort were also introduced to egg later. Of note, the association between egg introduction and Black race did not remain significant after multivariable analysis however the similarity in hazard ratios suggests that this may be because we were not powered for these sub-analyses.

There is data to suggest there are higher rates of allergy among Black children and that food allergy prevalence differs by race (17). While our study was not able to directly investigate the possible connection between delayed introduction and allergy prevalence by race, we hope further research will explore this. More research is also required to evaluate what factors may be affecting the age of introduction of allergens in Black and Asian children, so that allergy prevention initiatives can be effectively identified and utilized.

Our study does have several limitations. Guidelines have changed over the last several decades from delayed introduction (2000) to not delaying introduction (2008) to early introduction (2017, 2019) (3, 18). Of course, these changing guidelines don't lead to instantaneous awareness nor implementation on any date. Our analysis sought to evaluate the degree to which introduction is changing in the years since the 2017 guidelines, but we recognize this is a fluid process and that it often takes more than a year for new guidelines to be widely adopted. We did not capture the degree to which physicians and parents at the practice were aware of and implementing the 2017 NIAID guidelines. We also had fewer children in our post-guidelines group given our enrollment period. Our sample size was also limited for participants identifying as Black compared to other race categories, and our cohort was predominately white. This does limit the significance of our findings with respect to Black children and allergen introduction, but we hope it encourages further research to continue to evaluate the potential association between race and allergen introduction. Children in the subset analyzed (due to complete allergen introduction data) had a higher proportion of caregiver-reported eczema than the larger GMAP cohort did, and this may mean they were counseled more carefully about allergen introduction. However, this was not different between groups analyzed and does not change any of our reported associations with outcomes. It may mean that the true percentages of early introduction in the full cohort are even lower.

Current guidelines highlight the importance of early introduction of foods for the prevention of food allergy. Our work, however, illustrates the ongoing need for dissemination of guidelines for allergy prevention and may also identify populations of children who are at particular risk for later introduction (children with older siblings, children who are Black or Asian). Ongoing research to evaluate current practices for allergen introduction in the U.S. with a focus on disparities is needed.

## Data Availability

The raw data supporting the conclusions of this article will be made available by the authors, without undue reservation.
