# Supplementary material for: Factors influencing age of common allergen introduction in early childhood
Source: Front Pediatr. 2023 Jul 11;11:1207680. doi: 10.3389/fped.2023.1207680 (PMC10366355; doi:10.3389/fped.2023.1207680)
Supplement: Supplementary file 1 [file Table1.pdf]

Supplemental Table 1. Factors Associated with Time to Introduction of Peanut and Egg by Survival Analysis

		Univariable				Multivariable			
		Peanut		Egg		Peanut		Egg	
**Variable**	***n* (%)**	**HR [CI]**	***P***	**HR [CI]**	***P***	**HR [CI]**	***P***	**HR [CI]**	***P***
Female	232 (47%)	0.9 [0.74, 1.08]	0.3	0.88 [0.73, 1.06]	0.2	0.96 [0.78, 1.17]	0.7	0.93 [0.77, 1.13]	0.5
Race									
White	348 (70%)	—	—	—	—	—	—	—	—
Black	8 (1.6%)	**0.3 [0.14, 0.64]**	**0.002**	**0.42 [0.21, 0.86]**	**0.02**	**0.27 [0.11, 0.69]**	**0.006**	0.48 [0.21, 1.09]	0.08
Asian	84 (17%)	**0.66 [0.51, 0.86]**	**0.002**	**0.73 [0.57, 0.94]**	**0.02**	**0.64 [0.47, 0.86]**	**0.003**	**0.74 [0.55, 0.97]**	**0.03**
Other Race	48 (10%)	0.81 [0.59, 1.12]	0.2	0.82 [0.60, 1.12]	0.2	0.84 [0.60, 1.19]	0.3	0.87 [0.62, 1.21]	0.4
Hispanic or Latino	23 (4.7%)	0.99 [0.65, 1.50]	0.9	0.81 [0.52, 1.24]	0.3	0.89 [0.57, 1.39]	0.6	0.76 [0.49, 1.20]	0.2
Gestational Age									
>37 Weeks	448 (91%)	—	—	—	—	—	—	—	—
33-37 Weeks	43 (9%)	0.97 [0.69, 1.36]	0.8	0.87 [0.63, 1.20]	0.4	1.00 [0.70, 1.44]	1.0	0.83 [0.59, 1.17]	0.3
25-32 Weeks	3 (0.6%)	1.18 [0.38, 3.67]	0.8	1.39 [0.45, 4.33]	0.6	0.99 [0.31, 3.20]	1.0	1.11 [0.35, 3.55]	0.9
Eczema	250 (51%)	0.91 [0.76, 1.1]	0.3	0.91 [0.76, 1.09]	0.3	0.91 [0.74, 1.12]	0.4	0.91 [0.75, 1.11]	0.4
First Child	237 (48%)	**1.23 [1.02, 1.48]**	**0.03**	1.12 [0.94, 1.34]	0.2	**1.27 [1.04, 1.56]**	**0.02**	1.18 [0.97, 1.43]	0.1
IgE-FA	36 (7.3%)	0.81 [0.56, 1.18]	0.3	0.79 [0.55, 1.15]	0.2	0.69 [0.45, 1.05]	0.08	0.72 [0.48, 1.08]	0.1
Family History of IgE-FA	81 (16%)	1.03 [0.79, 1.35]	0.8	1.15 [0.90, 1.47]	0.3	0.86 [0.64, 1.15]	0.3	1.11 [0.85, 1.45]	0.4
FPIAP	88 (18%)	1.02 [0.80, 1.31]	0.9	0.91 [0.72, 1.16]	0.5	1.05 [0.80, 1.37]	0.7	0.91 [0.70, 1.18]	0.5
Post-2017 Guidelines	117 (24%)	1.16 [0.93, 1.43]	0.2	1.07 [0.87, 1.32]	0.5	1.21 [0.96, 1.53]	0.1	1.04 [0.83, 1.31]	0.7

* All variables above were used in model for multivariable analysis of allergen introduction
